# Two-photon excitation enables single-molecule detection of a fluorescent base analogue in DNA with high photostability

**DOI:** 10.1039/d5sc07287e

**Published:** 2026-01-01

**Authors:** Henry G. Sansom, Alexandra E. Bailie, Filippos Stefanou, Byron W. Purse, Anita C. Jones, Steven W. Magennis

**Affiliations:** a School of Chemistry, University of Glasgow, University Avenue Joseph Black Building Glasgow G12 8QQ UK steven.magennis@glasgow.ac.uk; b EaStCHEM School of Chemistry, The University of Edinburgh Joseph Black Building, David Brewster Road Edinburgh EH9 3FJ UK a.c.jones@ed.ac.uk; c Department of Chemistry and Biochemistry, San Diego State University San Diego CA 92182 USA bpurse@sdsu.edu

## Abstract

Fluorescent modification of nucleic acids using nucleobase analogues provides unique capabilities for imaging nucleic acids in cells and monitoring their conformational changes upon interaction with other biomolecules. The fluorescent nucleobase analogue ABN was shown recently to be the first base analogue to enable single-molecule detection of dsDNA, but its sensitivity to photobleaching under one-photon excitation required the use of oxygen scavenging reagents, and may limit future applications in live-cell imaging. Here, we show that two-photon excitation of ABN allows the stable detection of single DNA molecules in aqueous solution without antifade additives. The two-photon brightness of ABN in dsDNA exceeds by an order of magnitude that reported previously for any base analogue, and is comparable to that of the fluorescent protein EGFP, making it a promising candidate for the ultrasensitive imaging of nucleic acids in living cells and tissues.

## Abstract 2

DNA incorporating a fluorescent nucleobase analogue, ABN, has been detected at the single-molecule level using two-photon excitation. The high photostability, without the use of antifade reagents, opens the way for ultrasensitive *in vivo* imaging.

## Graphical abstract

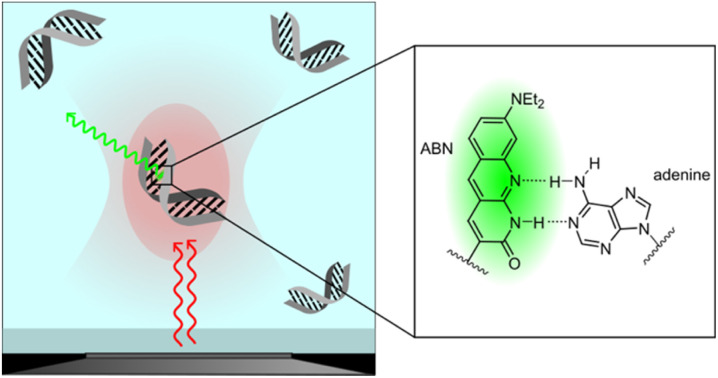

## Introduction

Fluorescent labelling of DNA and RNA is typically achieved through covalent attachment of bright extrinsic fluorophores, but these can perturb the nucleic acid structure^1^ and cannot be positioned precisely within the base sequence.^2^ Moreover, conventional fluorophore labelling can significantly alter the subcellular localization of biomolecules.^3^ An alternative is to employ a fluorescent base analogue (FBA) to replace a specific natural base in an oligonucleotide and preserve the native structure.^4–7^ Non-canonical nucleobases have enormous potential for biotechnological applications and such modifications can be compatible with normal cell processes.^8,9^ Live-cell imaging is a particularly exciting recent application of FBAs^10–12^ For example, FBAs have been used to image the uptake of translationally-competent mRNA into living cells, gain insights into riboswitch function, detect and measure the structural and dynamic consequences of DNA damage, and measure viscoelastic flow within the nucleolus.^4,10,12–19^ However, most FBAs are hampered by low brightness and a requirement for UV excitation, limiting their use in ultrasensitive applications.^20,21^

**Fig. 1:**
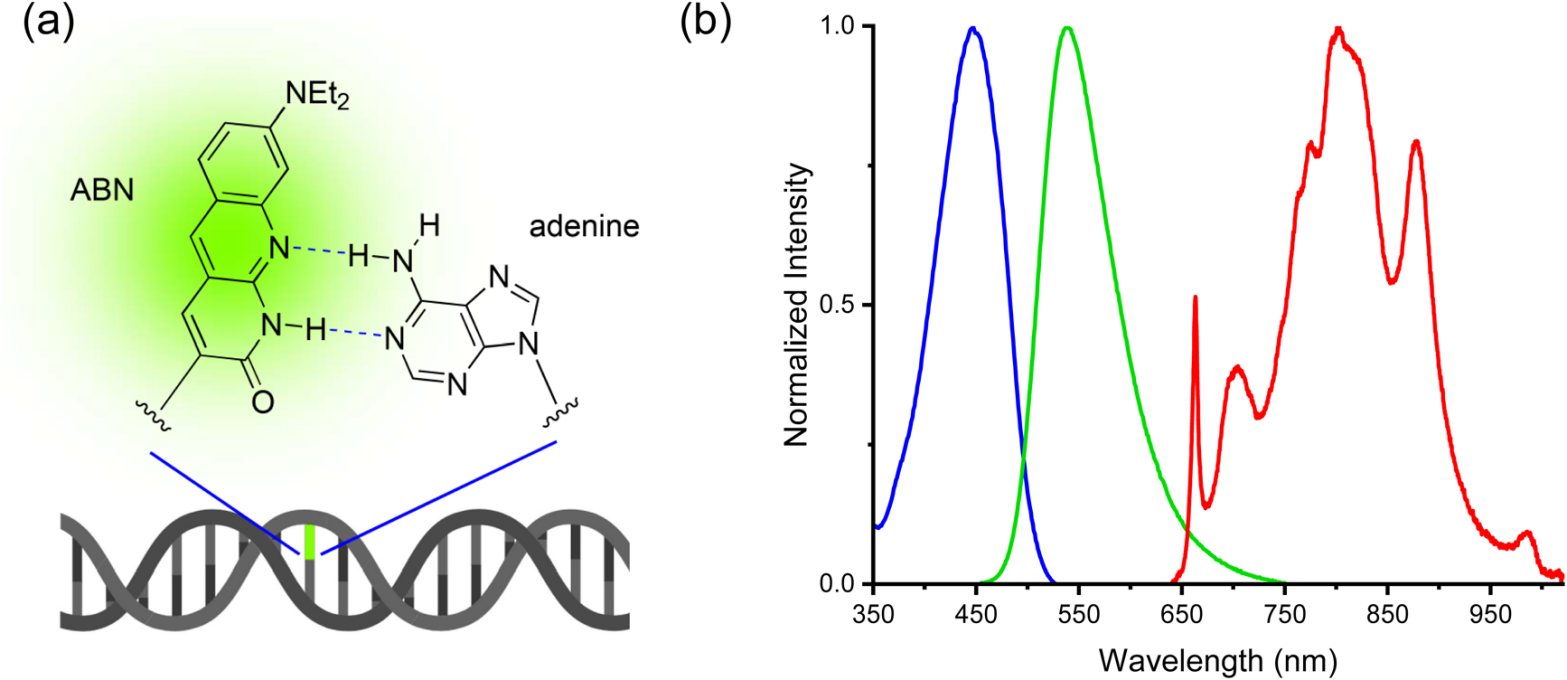
(a) The fluorescent base analogue ABN substituted for thymine in dsDNA (b) one-photon excitation (blue) and emission spectra (green) of ABN in dsDNA [sequence context GXC(A); see Fig. 2e for details], and the broadband laser spectrum used for two-photon excitation (red).

**Fig. 2:**
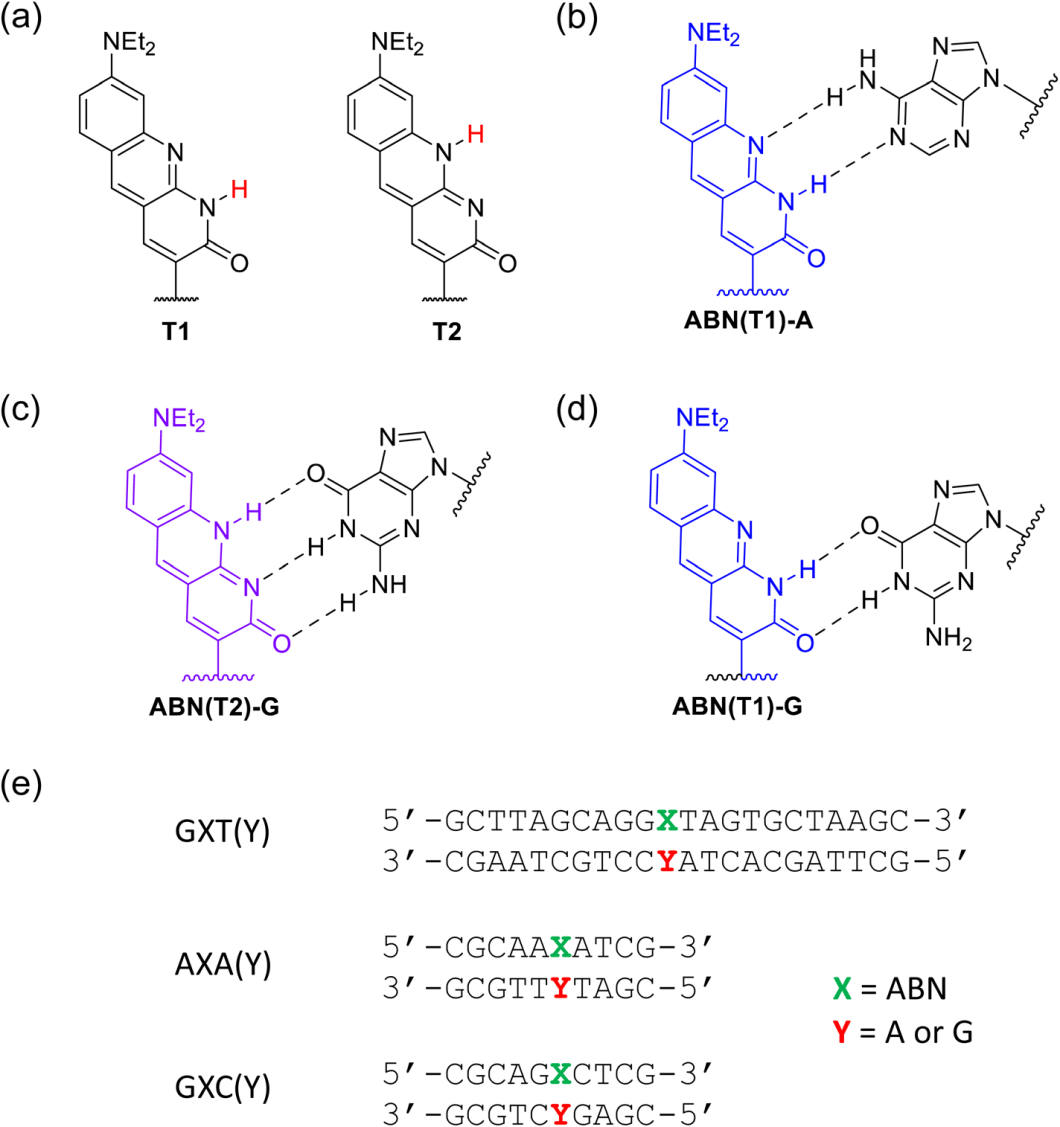
Tautomers of ABN and their base pairs with adenine (A) and guanine (G). (a) Thymine-like T1 and cytosine-like T2 tautomers of ABN nucleoside. (b) and (c) Watson–Crick base pairs T1:A and T2:G (d) wobble base pair T1:G (e) oligonucleotides are named according to the bases neighbouring ABN (denoted as *X*). Duplexes are named to denote the base that is paired with ABN in the complementary strand.

Single-molecule detection has, however, been achieved recently *via* one-photon (1P) excitation for ABN, a fluorescent pyrimidine analogue, both as a nucleoside^22^ and when incorporated into an oligonucleotide (Fig. 1a).^23^ ABN (8-(diethylamino)benzo[*b*][1,8]naphthyridin-2(1*H*)-one) was designed as a push–pull FBA dye to mimic key features of bright xanthene fluorophores such as rhodamine B. ABN is unique among FBAs in combining exceptional brightness with long-wavelength absorption, *λ*_max_ > 400 nm, and emission, *λ*_max_ > 500 nm, (Fig. 1b). It can exist in two tautomeric forms (Fig. 2a), a thymine-like tautomer (T1) and a cytosine-like tautomer (T2) (see summary in SI). The free nucleoside exists exclusively as T1, but in duplexes, the tautomeric populations of ABN depend on its base-pair partner (Fig. 2b–d), which can be either adenine (T1 in Watson–Crick base pair) or guanine (T2 in Watson–Crick base pair and minor population of T1 in wobble base pair).^23^ As demonstrated previously through base-pairing analyses, melting-temperature measurements, and CD spectroscopy, ABN induces some degree of localized perturbation in the DNA duplex but does not compromise Watson–Crick base pairing or the overall right-handed B-form structure.^22^ Single ABN oligonucleotides were detected previously using total internal reflection fluorescence (TIRF) 1P microscopy (representative 1P spectra for the nucleoside and duplexes are shown in Fig. S1 and S2).^23^ Oxygen-scavengers and Trolox improved the photostability of the surface-immobilised molecules, but fast ABN photobleaching was still evident, suggesting that photodegradation is not primarily a triplet-state process. Furthermore, although antifade agents are routinely employed *in vitro*, this approach is generally incompatible with *in vivo* measurements.^24^

Multiphoton excitation (MPE) of FBAs can potentially overcome the photophysical and photochemical limitations of 1P excitation, and has been a transformative tool for biological imaging.^25^ Advantages of MPE include a 3D-confined excitation volume, reduced out-of-focus photobleaching and autofluorescence, and increased penetration depth *in vivo*.^25,26^ Bulk multiphoton studies of FBAs have been reported,^27–33^ while ensemble two-photon (2P) imaging in cells has been demonstrated recently for an FBA incorporated in an antisense oligonucleotide.^34^ Previously we have demonstrated the detection of less than ten FBA-labelled oligonucleotides using pulse-shaped MPE.^35–37^ Here we show that ABN in DNA exhibits a 2P brightness that is as high as that of fluorescent proteins such as EBFP, ECYP and EGFP under similar excitation conditions.^38^ This advance has enabled the single-molecule detection of ABN-containing oligonucleotides without the use of antifade reagents, using 2P excitation.

## Results and discussion

Ensemble multiphoton spectroscopy was performed to quantify the efficiency of 2P excitation in terms of the 2P cross section (*σ*_2_), which is analogous to the 1P molar absorption coefficient and is measured in Goeppert-Mayer (GM) units (1 GM = 10^−50^ cm^4^ s).^38^ The experimental measurand is the 2P brightness, also measured in GM units, which is the product of the 2P cross section and the fluorescence quantum yield. The 2P cross section is derived by dividing the 2P brightness by the quantum yield, measured under 1P excitation. This rests on the assumption that the emitting population, and hence the quantum yield, is the same under 1P and 2P excitation. For all of the samples studied here, this assumption is supported by the close similarity of the emission spectra under the two excitation regimes, as shown in Fig. S3 and S4.

**Fig. 3:**
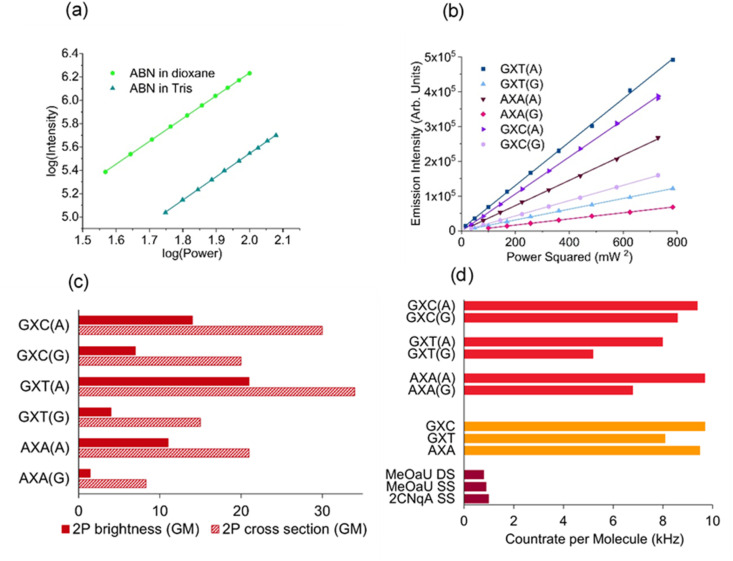
(a) Log–log plot of emission intensity *versus* 850 nm laser power for ABN nucleoside in dioxane and Tris buffer; the gradients are 1.96 and 2.00, respectively. (b) Plot of emission intensity *versus* the square of 890 nm laser power for the ABN-containing duplexes; the gradient is proportional to the 2P brightness. (c) The 2P brightness and 2P cross sections of the ABN-containing duplexes. (d) The countrate per molecule measured by FCS for the ABN-containing duplexes (red), single strands (orange) and FBA-containing oligos reported previously (dark red).^34,36^ (Plots (a) and (b) show triplicate measurements at each laser power, but points overlap too closely to be discerned).

Multiphoton spectroscopy of the ABN nucleoside, in dioxane and Tris, was performed at an excitation wavelength of 850 nm; the log–log plots of emission intensity *versus* laser power (Fig. 3a) confirm a 2P absorption process. The 2P brightness (Table 1) in Tris (4.7 GM) is much lower than that in dioxane (30 GM), due to both a lower quantum yield (Table S1) and a lower 2P cross section (Table 1).

**Table 1:** Two-photon brightness (*σ*_2_*ϕ*) and two-photon cross section (*σ*_2_) with *λ*_ex_ = 850 nm for the nucleoside and *λ*_ex_ = 890 nm for the duplexes. Cross section values were calculated using quantum yields measured at excitation wavelengths of 420 nm for the free nucleoside, and 440 nm for the oligonucleotides

Sample	*σ*_2_ *ϕ*/GM^a^	*σ*_2_/GM
Nucleoside in dioxane	30	35
Nucleoside in Tris	4.7	15
GXC(A)	14	30
GXC(G)	7.0	20
GXT(A)	21	34
GXT(G)	4.0	15
AXA(A)	11	21
AXA(G)	1.4	8.3

a Estimated uncertainty is 10%.

In both solvents we are measuring the cross section of the T1 tautomer (Fig. 2a), which is populated exclusively in the ground state (see SI).^23^ The 2P brightness of ABN in dioxane is almost a factor of two greater than the highest value reported previously for an FBA, 18 GM for DMA^th^aU in dioxane.^37^ More remarkable is the high 2P brightness retained by ABN when incorporated in duplex DNA.

The 2P brightness of the six duplexes (Fig. 2e) was measured at excitation wavelengths of 850, 870 and 890 nm (Table S2); the quadratic dependence of emission intensity on laser power confirmed 2P absorption (Fig. 3b and Table S3). In all cases, the highest brightness was observed at 890 nm (Fig. 3c and Table 1). When ABN is base-paired with A it displays notably higher 2P brightness than when paired with G (Fig. 3b, c and Table 1), due primarily to the lower quantum yields of the latter duplexes (Table S1), in which the more weakly emitting T2 is dominant (see SI for details).^23^

The 2P cross sections at 890 nm are somewhat higher for the ABN:A duplexes than for ABN:G (Fig. 3c and Table 1), attributed to the red shift in the absorption spectra of the latter (Fig. S2 and Table S1). We expect the 2P cross-section maximum to be close to 890 nm for ABN:A, but around 940 nm for ABN:G. For chromophores lacking a centre of symmetry, the spectral profiles for 2P and 1P absorption are often similar.^39,40^ Assuming this applies to ABN, *i.e.* the ratio of the cross sections at 940 nm and 890 nm is approximately equal to the ratio of the molar absorption coefficients at 470 nm and 445 nm, we estimate the maximum 2P cross-section values of ABN:G to be about 50% greater than the 890 nm values (Table S2), but they remain lower than those for the corresponding ABN:A.

For ABN:A we are measuring the cross section of the T1 tautomer, as for the free nucleoside, and its magnitude is not detrimentally affected by incorporation in the DNA environment. The values for GXT(A), 34 GM, and GXC(A), 30 GM, are close to that of the nucleoside in dioxane, 35 GM. The value for AXA(A), 20 GM, is somewhat lower, but remains higher than that of the nucleoside in Tris, 15 GM. For the ABN:G, the measured cross section is the average value for the combined population of T1 and T2 tautomers, weighted towards T2 (see SI for details). The decrease in cross section when changing the base-pair partner from A to G suggests that T2 has a lower cross section than T1; however, in these duplexes, T1 forms a wobble-base pair (Fig. 2d) rather than a Watson–Crick base pair (Fig. 2b), which may also affect the cross section. The 2P brightness of the ABN-containing duplexes greatly exceeds the highest value reported previously for an FBA in duplex DNA, which was 0.46 GM for pA.^35^ The large 2P cross sections and brightness for ABN in DNA (up to 34 and 21 GM, respectively) compare favourably to the archetype for *in vivo* labelling, the fluorescent proteins. Using similar excitation conditions, the 2P cross section/brightness, in units of GM, for EBFP, ECFP and EGFP are 13/9.2, 23/12 and 39/30, respectively.^38^

**Fig. 4:**
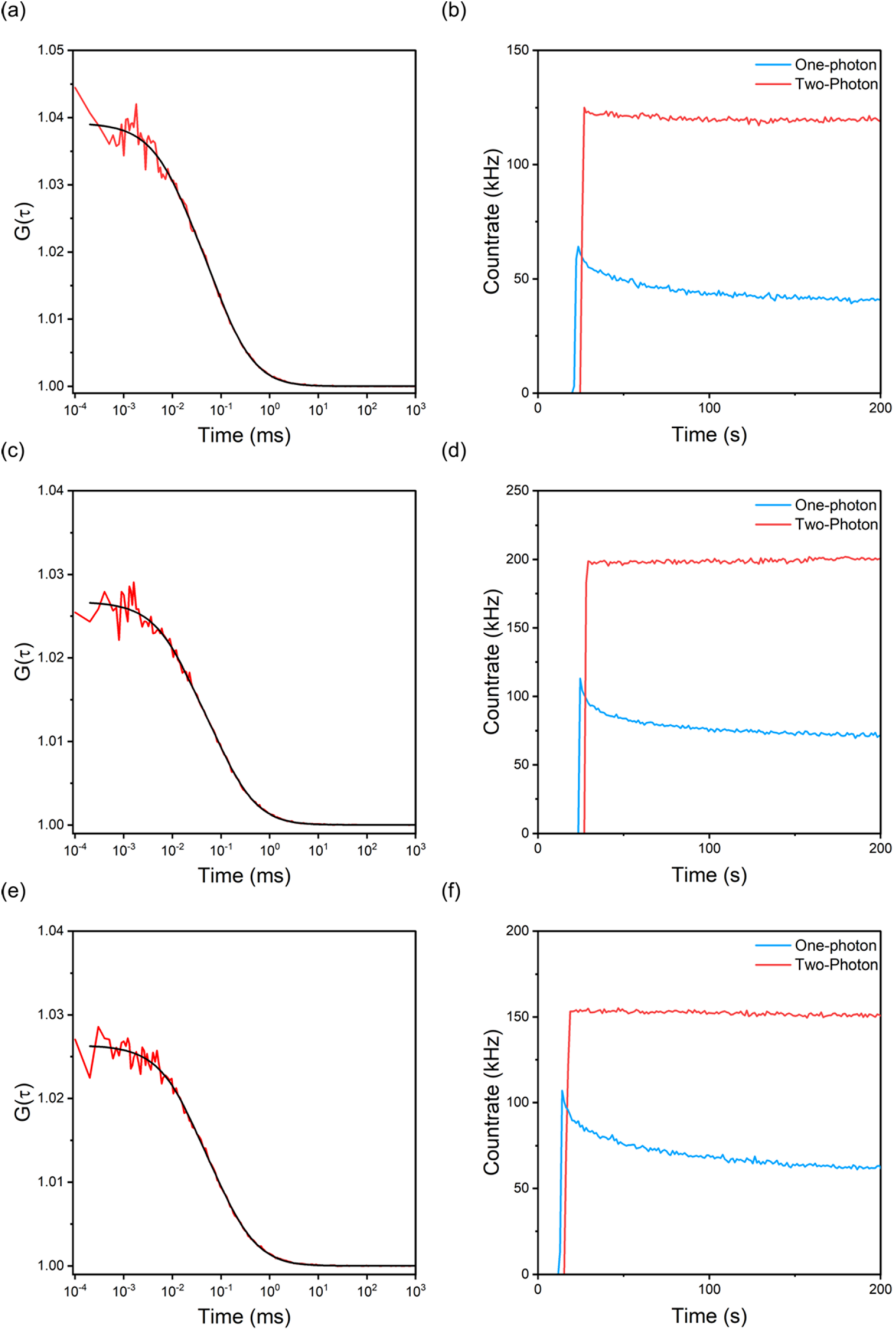
(a), (c) and (e) 2P FCS of 100 nM GXC, GXC(A) and GXC(G), respectively; the red line is the correlation, and the black line is the fit. (b), (d) and (f) Photostability of GXC, GXC(A) and GXC(G), respectively, under 1P (blue) and 2P (red) excitation; the traces have been offset in time for clarity.

Given their unprecedented brightness, we investigated the suitability of ABN-containing oligonucleotides for single-molecule detection in Tris buffer, without addition of antifade reagents, using fluorescence correlation spectroscopy (FCS). The excitation source was a pulse-shaped broadband Ti:sapphire laser centred at 800 nm with 220 nm bandwidth (Fig. 1b), giving compressed pulses of 7 fs FWHM at the sample with an average power of 11 mW.^41^ FCS data were fitted to a model for fluctuations due to 3D diffusion and the population of a dark state (eqn S2). As expected, the diffusion times increased after incorporation of the single strands in a duplex (Table S4). The FCS curves for GXC (Fig. 4a and c) are typical of the data for all samples (Fig. S5). Using the same instrumental setup, all sequences were measured to have higher countrates per molecule (CPM) than any previous FBA incorporated into oligonucleotides (Fig. 3d and Table S4).^34,36,41^ The highest CPM of 9.7 kHz was for the single strand GXC and the duplex AXA(A). This brightness is over three times that measured using 1P excitation (3 kHz)^23^ and is an order of magnitude greater than for oligonucleotides containing the FBAs 2CNqA^34^ and MeO^th^aU^36^ (Fig. 3d), and higher than the ribonucleoside of DMA^th^aU, which was detected previously at the single-molecule level.^37^ As observed at the ensemble level, the CPM is notably lower for ABN:G than ABN:A, but this trend correlates more closely with ensemble cross section than ensemble brightness. This observation is consistent with the wavelength profile of the broadband excitation, which results in preferential excitation of T1 in the ABN:G duplexes and hence more similar quantum yields for ABN:G and ABN:A in FCS measurements.

A major advantage of ABN is the absence of long-lived dark states,^22^ which are found in some other FBAs, including DMA^th^aU^37^ and MeO^th^aU.^36^ For the sequences measured here, ABN spends 22–28% of its time in a short-lived dark state, and this appears to be essentially independent of sequence context; including this dark state is necessary for an adequate fit (Fig. S6). We assign this dark state to a triplet state, based on experiments on the free ABN nucleoside using the triplet-state quencher mercaptoethylamine (MEA). We selected MEA after testing the 1P photostability of the ABN nucleoside in the presence of either a reductant (*n*-propyl gallate (nPG) or Trolox) or MEA. For 1P studies, these were tested in combination with the “gloxy” oxygen scavenging system in Tris buffer (Fig. S7a). The best antifade buffer was the one containing MEA which gave strong suppression of photobleaching. We also performed 2P FCS in the presence and absence of MEA without the oxygen-scavenging components (Fig. S7b and c). There was an increase in the number of molecules in the focus from 14.1 ± 0.7 to 17.8 ± 0.4 coupled with an increase in the diffusion time from 78 ± 6 µs to 110 ± 3 µs. The triplet state fraction was unchanged; however, there was a reduction in triplet state lifetime from 17 ± 4 µs to 9 ± 2 µs. There was also a 15% increase in CPM, from 8.4 ± 0.5 kHz to 9.7 ± 0.4 kHz. The increase in diffusion time and number of molecules in the focus indicates an increase in the effective volume. The increase in the volume can be explained by a reduction of the in-focus photobleaching, in line with the reduction in the triplet state lifetime.^42^

To compare with 2P FCS, we recorded 1P FCS measurements using an excitation wavelength of 405 nm, which is the 1P equivalent of that used for 2P excitation (Fig. 1b). While 2P excitation produced stable traces with a constant countrate (Fig. 4b, d, f and S8–S10), the optimal 1P excitation conditions always resulted in lower initial emission intensity and rapid photobleaching (Fig. 4b, d, f and S8–S10). The enhancement of photostability under 2P excitation is independent of the identity (or presence) of the base-pair partner and sequence context, indicating that this is an intrinsic property of the ABN fluorophore and that both tautomers show similar behaviour. Increased photostability following 2P excitation has also been observed for the FBAs, tC,^29,41^ pA^35^ and 2CNqA.^34^ In those previous examples, and in the present case, the same excited electronic state (S_1_) is expected to be populated initially by 1P and 2P absorption, at the respective wavelengths used. This implies that photobleaching occurs *via* subsequent excited-state absorption, in either the singlet or triplet manifold. The 1P photobleaching rates presented above suggest that triplet states play a major role in the process in ABN. This would support a mechanism in which absorption of near-infrared photons in the triplet manifold depletes the triplet state population by inducing reverse intersystem crossing, as has been proposed previously for fluorescent proteins.^43^ We are aware of two previous studies where enhanced bleaching following 2P excitation has been observed for rhodamine flurophores.^44,45^ However, in these cases, different excited states were populated by the different excitation regimes; 1P excitation at ∼500 nm populated S_1,_ whereas 2P excitation at ∼800 nm populated a higher excited singlet state. A detailed examination of the photobleaching kinetics of rhodamine 6G^42^ indicated that the mechanism is photolysis from higher excited states (2-step photolysis). This is consistent with the behaviour of ABN (and other FBAs), where 2P excitation into S_1_ increases photostability by impeding secondary excitation to higher excited states.

**Fig. 5:**
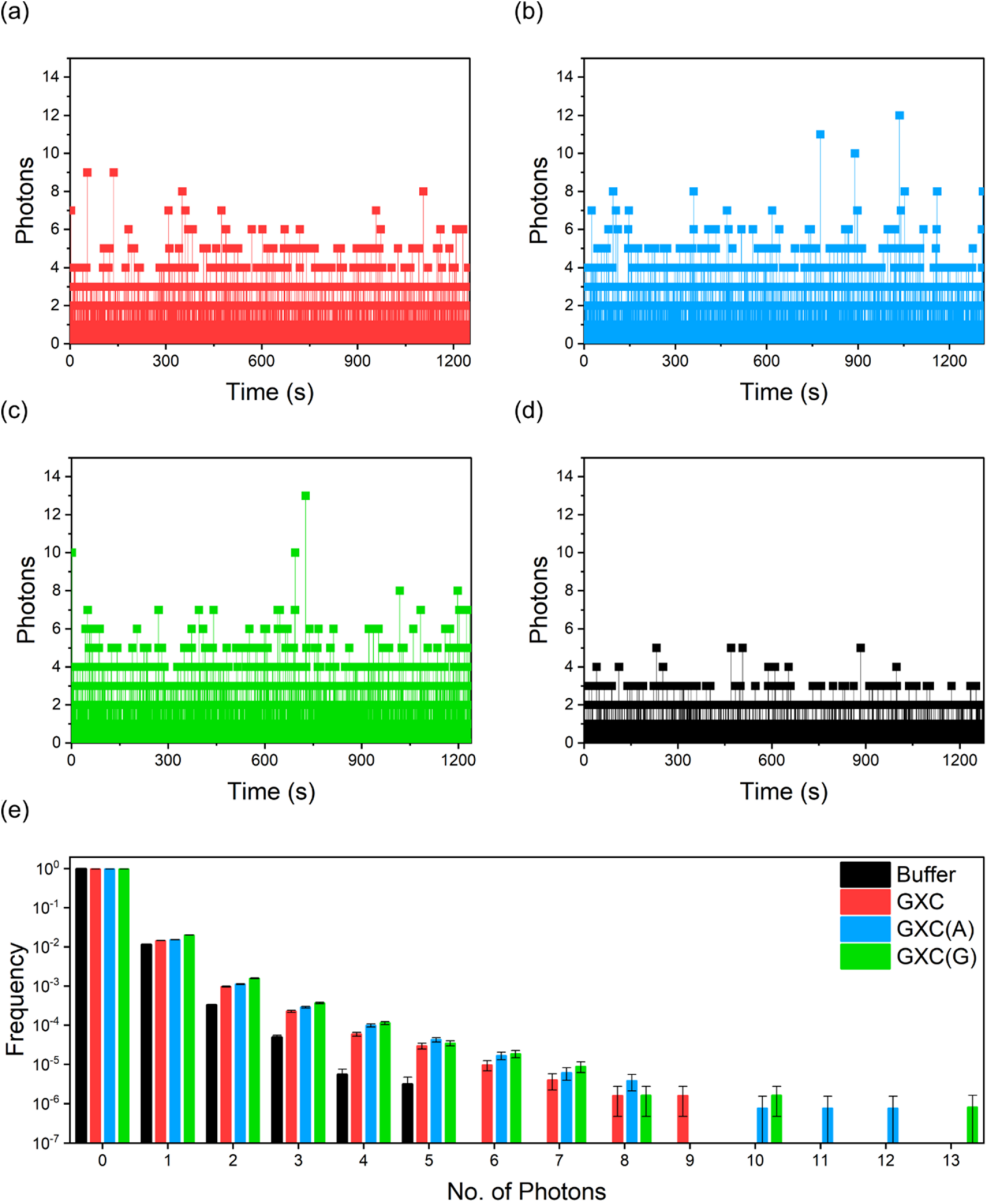
Single-molecule burst analysis of ABN oligos. (a) GXC (b) GXC(A) (c) GXC(G) (d) buffer only; excitation power was 11 mW with 1 ms bins (e) photon-counting histograms for the data in (a)–(d).

Given their high brightness and photostability under 2P excitation, we performed a single-molecule burst analysis of GXC and its corresponding duplexes GXC(A) and GXC(G) as they diffused through the laser focus. The ∼10 pM solutions result in an average of ∼0.001 molecules in the focus. Single-molecule bursts were observed for all three samples (Fig. 5a–c), above the buffer-only background (Fig. 5d). The histogram of photon-counting events (Fig. 5e),^46^ shows that the largest bursts per 1 ms time bin were 9, 12 and 13 for GXC, GXC(A) and GXC(G), respectively. The magnitudes of the bursts correlate with the brightness measured *via* FCS (Table S4).

## Conclusion

We have shown that detection of a single FBA in a single oligonucleotide is possible with 2P excitation, owing to ABN's 2P brightness, which is on a par with key biological fluorophores such as EGFP. Furthermore, we have shown 2P excitation into the S_1_ state greatly reduces the rate of photobleaching, compared with the equivalent 1P excitation process. The combination of the unique optical properties of MPE, and the exceptional brightness and photostability of ABN-labelled DNA upon 2P excitation, without the need for antifade reagents, makes this a promising future approach to the ultrasensitive and single-molecule imaging of FBA-labelled nucleic acids *in vivo*.

## Author contributions

All authors were involved in the design of the research. A. E. B. performed the ensemble experiments. H. G. S. performed the single-molecule experiments. F. S. performed the ensemble antifade experiments, with assistance from H. G. S. A. C. J. and S. W. M. supervised the project. The data were analysed by A. E. B., H. G. S., A. C. J. and S. W. M. Labelled oligonucleotides were provided by B. W. P. All authors contributed to the writing and editing of the manuscript.

## Conflicts of interest

There are no conflicts to declare.

## Supplementary Material

SC-OLF-D5SC07287E-s001

## Data Availability

Supplementary information (SI): a summary of the one-photon photophysics of ABN and ABN-labelled DNA duplexes, experimental procedures, and additional photophysical data. See DOI: https://doi.org/10.1039/d5sc07287e.
