# Supplementary material for: Two-photon excitation enables single-molecule detection of a fluorescent base analogue in DNA with high photostability
Source: Chem Sci. 2026 Jan 1;17(8):4174–82. doi: 10.1039/d5sc07287e (PMC12777068; doi:10.1039/d5sc07287e)
Supplement: SC-017-D5SC07287E-s001 [file SC-017-D5SC07287E-s001.pdf]

Supplementary Information (SI) for Chemical Science.
This journal is © The Royal Society of Chemistry 2026

SUPPLEMENTARY INFORMATION

Two-photon excitation enables single-molecule detection of a fluorescent base analogue in DNA with high photostability

Henry G. Sansom,[1,a] Alexandra E. Bailie,[2,a] Filippos Stefanou,[1] Byron W. Purse,[3,*] Anita C. Jones[2,*] and Steven W. Magennis[1,*]

[1] School of Chemistry, University of Glasgow, Joseph Black Building, University Avenue, Glasgow G12 8QQ, UK

[2] EaStCHEM School of Chemistry, The University of Edinburgh, Joseph Black Building, David Brewster Road, Edinburgh, EH9 3FJ UK

[3] Department of Chemistry and Biochemistry and the Viral Information Institute, San Diego State University, San Diego, CA 92182, USA.

*To whom correspondence should be addressed. Email: bpurse@sdsu.edu, a.c.jones@ed.ac.uk and steven.magennis@glasgow.ac.uk

[a] H.G.S. and A.E.B. contributed equally to this work.

Contents

1. Summary of 1P Photophysics of ABN and ABN-labelled DNA Duplexes........................2

2. Materials and Methods..3

2.1. Oligonucleotides..3

2.2. Ensemble 2P Spectroscopy..3

2.3. 2P Microscopy..4

3. Supplementary Tables...6

4. Supplementary Figures..9

5. References..17

1. Summary of 1P Photophysics of ABN and ABN-labelled DNA Duplexes

Our previous studies of the ensemble photophysics of ABN under one-photon excitation[1-2] showed that it can exist in two tautomeric forms, a thymine-like tautomer (T1) and a cytosine-like tautomer (T2) as shown in Figure 1a; it also exhibits excited-state tautomerization. As the free nucleoside, ABN exists only as the thymine-like T1 tautomer in the ground-state, but excited-state intramolecular proton transfer generates the cytosine-like T2 tautomer. Thus, the absorption spectrum of the nucleoside is due to the T1 tautomer, while the emission spectrum has contributions from both T1 and T2. T2 emits at slightly longer wavelength than T1, but the spectra overlap and cannot be resolved; approximately 80% of the emission intensity is due to T1. The absorption, excitation and emission spectra of the nucleoside, under 1P-excitation, are shown in Figure S1.

In duplex oligonucleotides, the tautomeric populations of ABN depend on its base-pair partner (Figure 1b-d). When base-paired with A, ABN exists as the T1 tautomer, forming a Watson–Crick-like hydrogen-bonding pattern (Figure 1b). T1 in this base pair undergoes excited-state double proton transfer to form a tautomeric base pair between T2 and the imino form of A, as shown in Scheme S1.

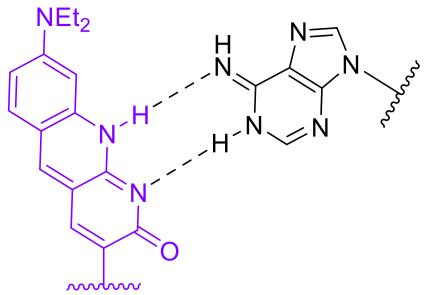

ABN(T2)-A (imino)

Scheme S1. Structure of the tautomeric base pair formed by excited-state double proton transfer from the Watson-Crick base pair T1:A.

The absorption and emission characteristics of ABN paired with A (ABN:A) thus resemble those of the free nucleoside; absorption due to T1 and emission from both T1 and T2, with T1 predominant. When base-paired with G, ABN exists predominantly as T2, to form Watson–Crick-like base pairs with G (Figure 1c). A minor fraction remains as T1, forming a wobble base pair (Figure 1d). Thus when ABN is paired with G (ABN:G), both T2 and T1 are excited directly and contribute to the absorption spectrum, with T2 predominant; the emission spectrum is also dominated by T2. The spectra of the duplex oligonucleotides are exemplified by those shown for GXC(A) and GXC(G) in

Figure S2. The effects of base-pair partner on the photophysical properties of the duplexes are summarised in Table S1. As a consequence of the different ground-state tautomer populations, the absorption spectra of the ABN:G duplexes are red-shifted relative to those of the ABN:A duplexes. The difference in excited-state tautomer populations results in a blue shift of the emission of ABN:G duplexes relative to ABN:A, and substantially lower quantum yields for the former.

2. Materials and Methods

2.1. Oligonucleotides

ABN-containing sequences and complementary strands were supplied by the lab of Byron Purse. Methods of synthesis of ABN and the ABN-containing oligonucleotides have been reported previously.[1-2] Complementary DNA sequences were purchased from Integrated DNA Technologies, Inc. (San Diego, CA).

2.2. Ensemble 2P Spectroscopy

Sample Preparation. The free nucleoside was dissolved in 1,4-dioxane (99.8 %, extra dry, Acros Organics) or Tris buffer (20 mM Tris with 150 mM NaCl, pH 7.5). The same Tris buffer was used to dissolve ABN-containing oligonucleotides. Duplex oligonucleotides were annealed by combining each modified single strand with 10% excess of its complementary strand at RT followed by heating to 95 °C and leaving to cool overnight. Absorption spectroscopy was used to confirm annealing.

Method. The excitation source was the fundamental output of a mode-locked Ti:sapphire laser (Coherent Mira pumped by Coherent Verdi), consisting of pulses of duration ~ 150 fs at a repetition rate of 76 MHz. A variable reflective neutral density filter was used to attenuate the excitation beam, which then passed through a dichroic mirror (Semrock Brightline FF735-Di02) and was focused by a 10x objective into the sample solution, contained in a 1-cm path-length cuvette. Fluorescence emission was collected by the same objective, reflected from the dichroic mirror, passed through a shortpass filter (Semrock Brightline FF01-720/SP-25) and detected by a fibre-coupled spectrometer (Ocean Optics USB2000+). Spectra were measured in triplicate and corrected for the wavelength response of the spectrometer. The incident laser power was measured using a Coherent FieldMaster power meter. Two-photon cross-sections were measured relative to Coumarin 153 in DMSO. Integrated emission intensity was measured in triplicate at 8-10 different incident laser powers.

The 2P brightness ($\sigma_2\phi$) of the sample, relative to that of the C153 reference, was determined from the gradient of a plot of integrated emission intensity versus the square of the incident laser power (exemplified in Figure 3(b) in the main text), as expressed by Equation S1. The 2P cross section was

then obtained by dividing the value of the 2P brightness by the quantum yield, measured under 1P excitation at the equivalent wavelength.

$$\frac{\sigma_2^{\mathrm{S}}\phi^{\mathrm{S}}}{\sigma_2^{\mathrm{R}}\phi^{\mathrm{R}}} = \frac{\eta^{\mathrm{R}} n^{\mathrm{S}} C^{\mathrm{R}} F^{\mathrm{S}} \langle P^{\mathrm{R}}\rangle^2}{\eta^{S} n^{\mathrm{R}} C^{\mathrm{S}} F^{\mathrm{R}} \langle P^{\mathrm{S}}\rangle^2} \qquad \text{(S1)}$$

where ϕ is the quantum yield of fluorescence, η is a correction term that accounts for the wavelength-dependent efficiency of fluorescence detection, n is the refractive index of the solvent, C is the concentration, F is the integrated fluorescence intensity from the recorded spectrum, $\langle P\rangle$ is the average laser power, and the superscripts S and R refer to either the sample or reference.

2.3. 2P Microscopy

Sample Preparation. The samples were initially dissolved in ultrapure water to a concentration of 100 µM before dilution in Tris buffer (20 mM Tris, 150 mM NaCl, pH 7.5) for measurement.

Duplexes were annealed in a 1:2 donor to complement ratio and heated to 95°C before being allowed to cool overnight.

Tris buffer (20 mM Tris, 150 mM NaCl) was prepared using ultrapure water, trimza base (Merck, BioUltra), trimza HCl (Merck, BioUltra), and NaCl (Merck, BioUltra). The pH was adjusted using 5 M HCl. To remove fluorescent impurities, buffer solutions were stirred with activated charcoal (Darco 4-12 mesh, Merck) and filtered through a 0.22 µm syringe driven filter before use.

Methods. The two-photon microscope is a homebuilt pulse-shaper assisted setup with an ultra-broadband Ti:Sapphire laser as light source. The broadband Ti:Sapphire laser had a repetition rate of 80 MHz and a spectrum centred on 800 nm with a FWHM of 135 nm (Vitara UBB, Coherent). The compressed pulses from the oscillator had a duration of 10 fs. To compensate for dispersion in the objective the pulses were compressed using the MIIPS method with a pulse shaper (Biophotonics, MIIPS-Box 640). The beam was subsequently focused onto the sample by a 60x water-immersion objective (UPlanSApo, Olympus). The pulses at the sample had a duration of 7 fs. The sample was placed on a cover slip (Menzel Gläser #1.5 thickness, Thermo Scientific). The solution temperature was controlled (22 ± 1°C) with an incubator (Live Cell Instrument, CU-501). Sample fluorescence was collected by the same objective and transmitted through a dichroic mirror (Chroma 675dcspxr) and a short-pass filter (Semrock FF01-650/SP). An additional bandpass filter was added to increase the

single-to-background ratio (FF01-565/133, Semrock). The emission signal was then split by a polarising beamsplitter cube into parallel and perpendicular channels and detected by two avalanche photodiodes (MPD PDM 50c and MPD $PD-050-CTB). In Figure 4 of the main text, the signals for parallel and perpendicular polarisation channels have been added together; in Figures S8-10, the two channels are shown separately. For the photostability and multichannel scalar measurements, two different avalanche photodiodes (APDs) were used (Hamamatsu, C11202-050), with lower dark counts and increased quantum efficiency. For fluorescence correlation spectroscopy (FCS), the signals from the APDs were cross-correlated using a hardware correlator (ALV-7002, ALV GmbH). For MCS experiments the signals from the SPADs first passed through a router (HRT-82, Becker and Hickl) before being detected by a photon-counting card (SPC-130, Becker and Hickl).

The photon-counting histogram (PCH) was generated from the MCS traces. Error bars in the PCH represent the statistical accuracy of the PCH determined by $\sigma = \sqrt{N(p(n)\big(1-p(n)\big)}$, where N is the total number of bins in the measurement and $p(n)$ is the relative frequency of an n-photon event.[3]

FCS curves were fitted to a model for diffusion through a 3D Gaussian focal volume along with an additional triplet state:

$$G(t) = \frac{\left(1-\frac{I_B}{S}\right)^2}{\sqrt{8}N(1-T)}\left(1+\frac{t}{\tau_D}\right)^{-1}\left(1+\frac{t}{k^2\tau_D}\right)^{-\frac{1}{2}} * \left[1-T+Texp\left(-\frac{t}{\tau_{triplet}}\right)\right]+1 \tag{S2}$$

Equation S2 takes into account the intensity of the background, I_B, and the signal, S. N is the number of molecules in the focus, τ_D is the characteristic diffusion time and $k = z_0/\omega_0$, where z_0 and ω_0 are the height and waist of the excitation volume, respectively.[4] T is the triplet state fraction and $\tau_{triplet}$ is the triplet state lifetime. The value of k was determined from a measurement of rhodamine 110 performed on the same day as the experiments on the oligonucleotides. For the experiments in this work, the value of k varied only slightly between 4.13 and 4.35. The rest of the parameters are extracted from the fits to the correlation curve.

For the comparative photostability measurements the excitation source was changed to a 405 nm CW diode laser (Cobolt 06-MLD, Hübner Photonics), and the dichroic (T412lpxt-UF1, Chroma) was

changed. A 100 µm pinhole was added to maintain confocality. Due to the difference in detection volume, the samples were diluted from 100 nM to 10 nM to maintain the same number of molecules in the focus.

3. Supplementary Tables

Table S1. One-photon photophysical properties of ABN nucleoside and ABN-containing duplex oligonucleotides: wavelengths of absorption and emission maxima and fluorescence quantum yields.[1, 2]

Sample	λ_{abs} max	λ_{em} max	φ^a
**ABN (tris)**	442	543	0.30
**ABN (dioxane)**	420	478	0.87
**GXC(A)**	450	538	0.48
**GXC(G)**	470	531	0.34
**GXT(A)**	440	533	0.61
**GXT(G)**	470	519	0.26
**AXA(A)**	445	530	0.54
**AXA(G)**	470	521	0.17

[a] The quantum yields of ABN in Tris and dioxane were measured at the absorption maxima of 440 and 420 nm, respectively. Quantum yields of oligos were measured at an excitation wavelength of 440 nm. Estimated uncertainty in quantum yield values is ±10 %.

Table S2. Two-photon cross-section values of duplex oligonucleotides measured at three excitation wavelengths. The estimated maximum cross-sections at 940 nm for ABN:G duplexes, based on the 1P absorption profiles, are also given.

Sample	σ^a /GM			Estimated σ_{max} /GM
	850 nm	870 nm	890 nm	940 nm
**GXC(A)**	20	22	30	-
**GXC(G)**	11	15	20	27
**GXT(A)**	25	26	34	-
**GXT(G)**	9.0	9.0	15	23
**AXA(A)**	15	16	21	-
**AXA(G)**	5.2	6.4	8.3	13

[a] Estimated uncertainty is 10%.

Table S3. Gradients of the plots of log(intensity) versus log (power) for two-photon excitation of the duplex oligonucleotides at 850, 870 and 890 nm.

**Sample**	**Gradient of log-log plot**		
	**850 nm**	**870 nm**	**890 nm**
**GXC(A)**	1.99	1.91	2.09
**GXC(G)**	1.99	1.98	2.05
**GXT(A)**	1.87	1.92	1.84
**GXT(G)**	1.95	2.01	1.97
**AXA(A)**	1.95	1.93	1.98
**AXA(T)**	2.01	2.01	2.11

Table S4. Two-photon fit parameters from FCS measurements of the ABN oligonucleotides (100 nM in Tris buffer; 11 mW excitation power).

Sample	CPM (kHz)	Diffusion Time (µs)	Triplet State Fraction	Triplet State Lifetime (µs)	N
**GXT**	8.1 ± 0.2	112 ± 2.5	0.24 ± 0.01	19 ± 4.4	15.7 ± 0.2
**GXT(A)**	8.0 ± 0.2	152 ± 5	0.28 ± <0.005	22 ± 2.1	19.8 ± 0.6
**GXT(G)**	5.2 ± 0.3	180 ± 18	0.26 ± 0.03	24 ± 10.1	25.8 ± 2.7
**AXA**	9.5 ± 0.1	100 ± 3	0.24 ± 0.02	16 ± 2.5	7.6 ± 0.3
**AXA(A)**	9.7 ± 0.1	123 ± 2	0.25 ± 0.01	17 ± 1.5	14.3 ± 0.4
**AXA(G)**	6.8 ± 0.3	120 ± 2.8	0.27 ± 0.01	16 ± 1.2	10.0 ± 0.5
**GXC**	9.7 ± 0.5	80 ± 6.7	0.22 ± 0.03	17 ± 5.7	11.9 ± 0.9
**GXC(A)**	9.4 ± 0.3	88 ± 3.6	0.24 ± 0.02	15 ± 2.7	17.6 ± 0.6
**GXC(G)**	8.6 ± 0.3	86 ± 4.6	0.21 ± 0.01	14 ± 6.1	16.5 ± 0.5

4. Supplementary Figures

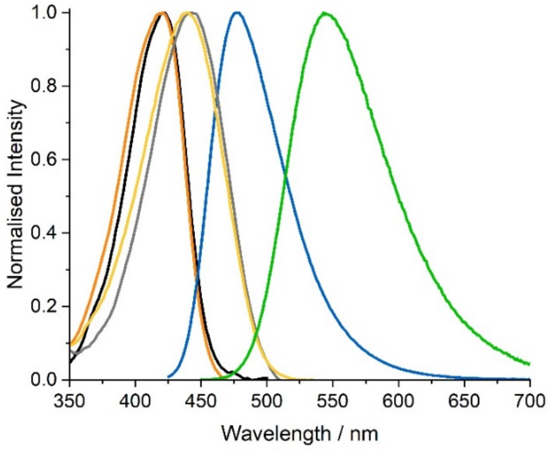

Figure S1. Normalised absorption, excitation and emission spectra of ABN nucleoside in dioxane and Tris.[2] Spectra in dioxane: absorption (black), excitation (orange) at emission wavelength 475 nm, emission (blue) at excitation wavelength 420 nm. Spectra in Tris: absorption (grey), excitation (yellow) at emission wavelength 540 nm, emission (green) at excitation wavelength 440 nm.

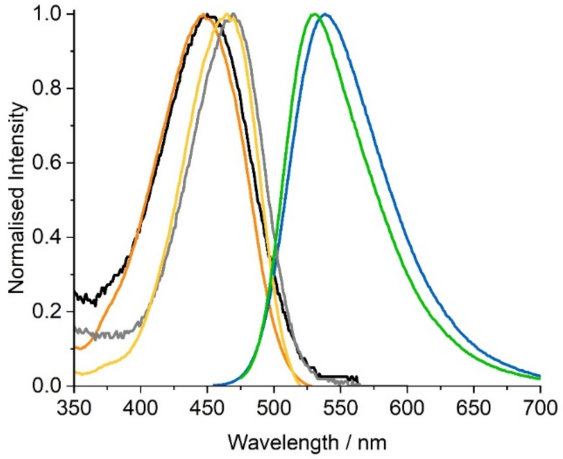

Figure S2. Normalised absorption, excitation and emission spectra of ABN in duplex oligonucleotides GXC(A) and GXC(G).[2] Spectra of GXC(A): absorption (black), excitation (orange) at emission wavelength 526 nm, emission (blue) at excitation wavelength 445 nm. Spectra of GXC(G): absorption (grey), excitation (yellow) at emission wavelength 520 nm, emission (green) at excitation wavelength 470 nm.

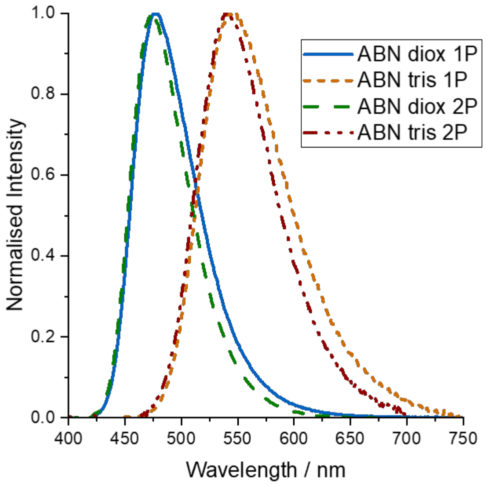

Figure S3. Comparison of the emission spectra recorded under one-photon (1P) and two-photon (2P) excitation for the free ABN nucleoside in dioxane and Tris buffer solutions.

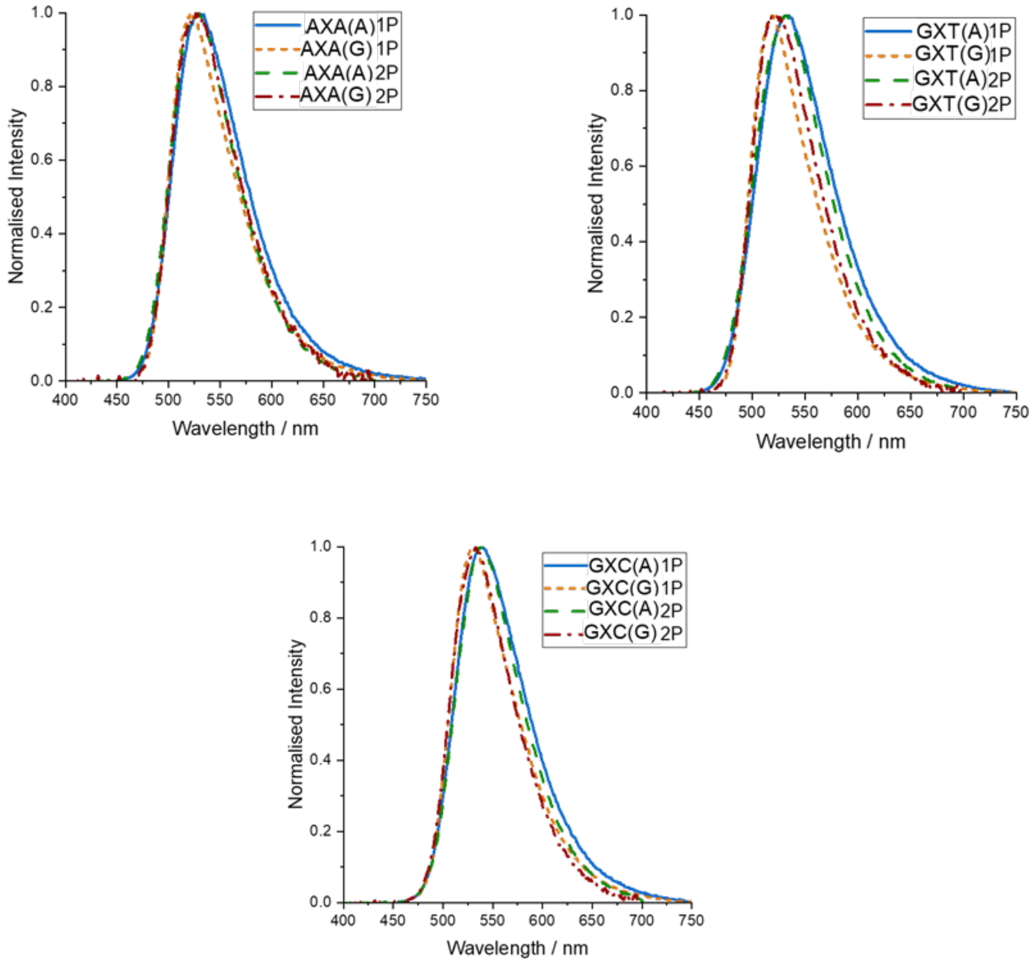

Figure S4. Comparison of the emission spectra recorded under one-photon (1P) and two-photon (2P) excitation for the ABN-containing duplex oligonucleotides.

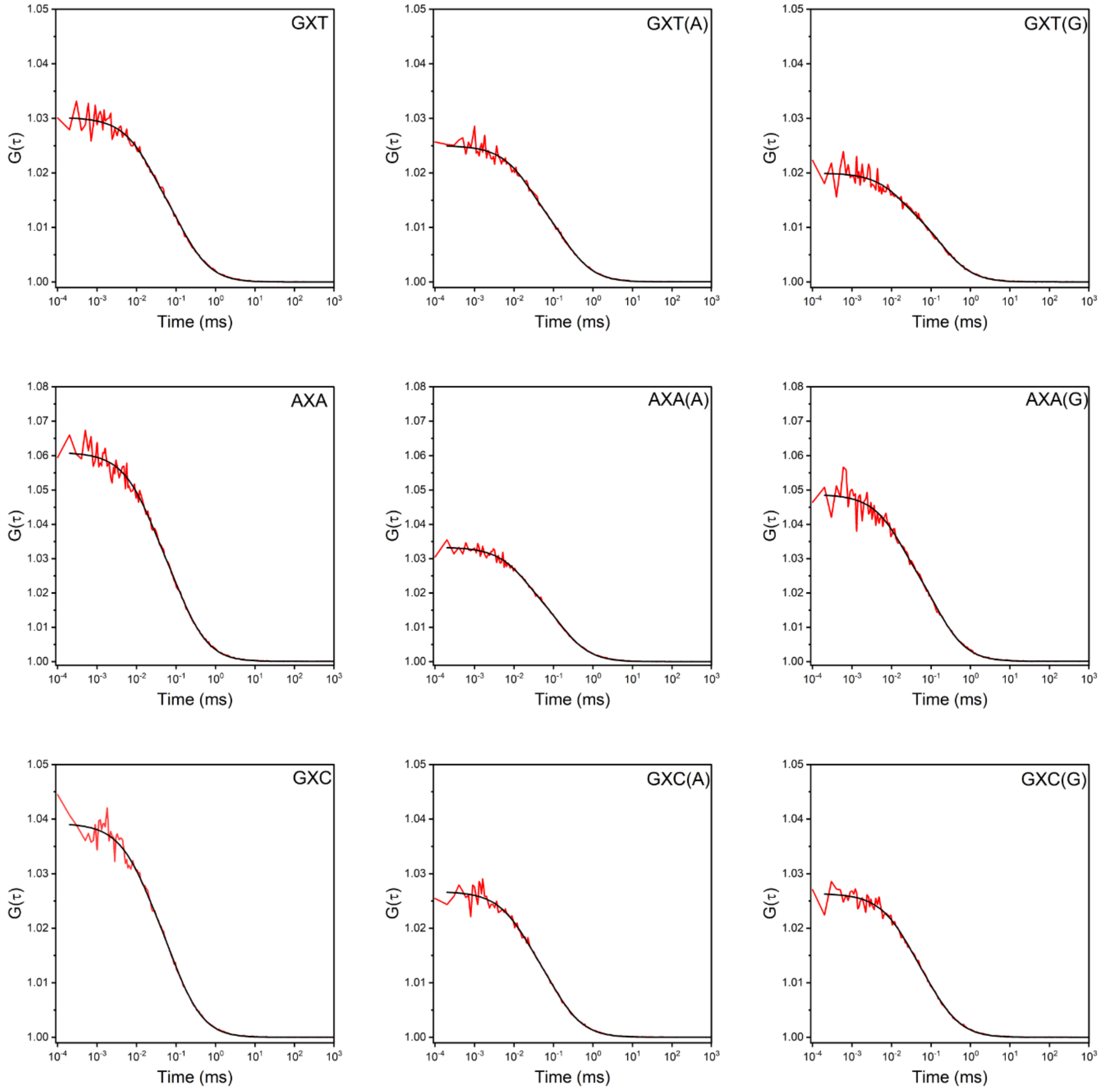

Figure S5. FCS curves of 100 nM samples of ABN-containing DNA single strands and duplexes. Excitation power was 11 mW.

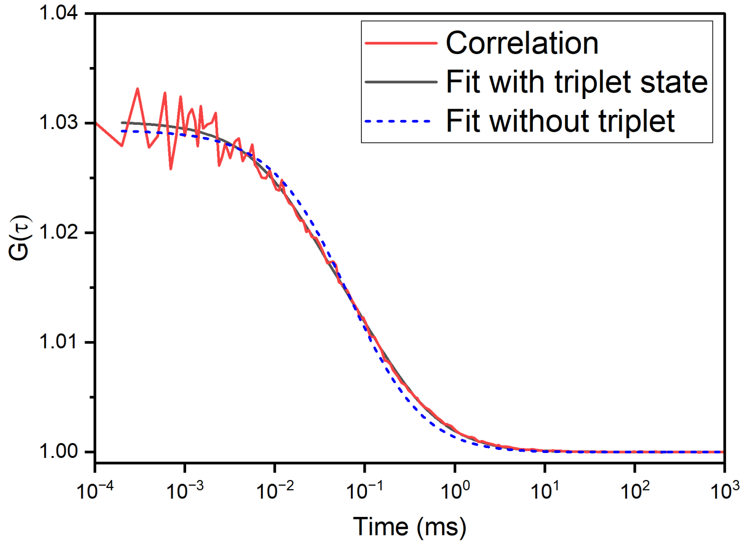

Figure S6. FCS data (red) for sample GXT showing fits to a model of 3D diffusion with (black) and without (blue) an additional triplet state. This is representative of fits for all ssDNA and dsDNA samples.

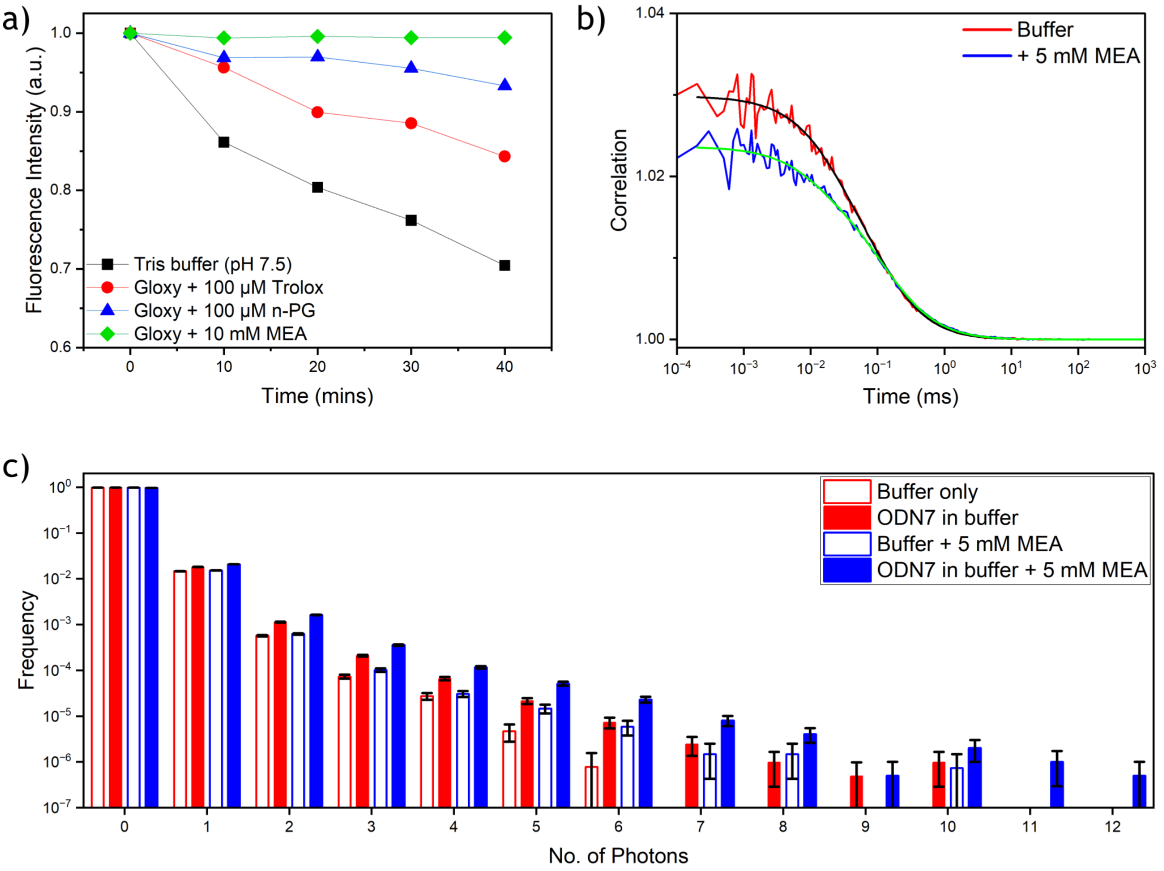

Figure S7. (a) Comparison of the photostability of the ABN nucleoside (1 µM) under continuous 1P excitation in the presence of antifade reagents: the reductants n-propyl gallate (nPG) and Trolox, and the triplet-state quencher mercaptoethylamine (MEA). All were tested in combination with the "gloxy" oxygen scavenging system (0.05 µM glucose oxidase and 0.8 µM catalase) in Tris buffer (20 mM Tris, 150 mM NaCl, pH 7.5) containing 6% w/v glucose. (b) 2P FCS of 100 nM GXC in Tris buffer before (red) and after (blue) the addition of 5 mM MEA. (c) 2P photon-counting histograms for Tris buffer or 10 pM GXC in Tris buffer, with and without 5mM MEA. Average power for 2P FCS was 11 mW.

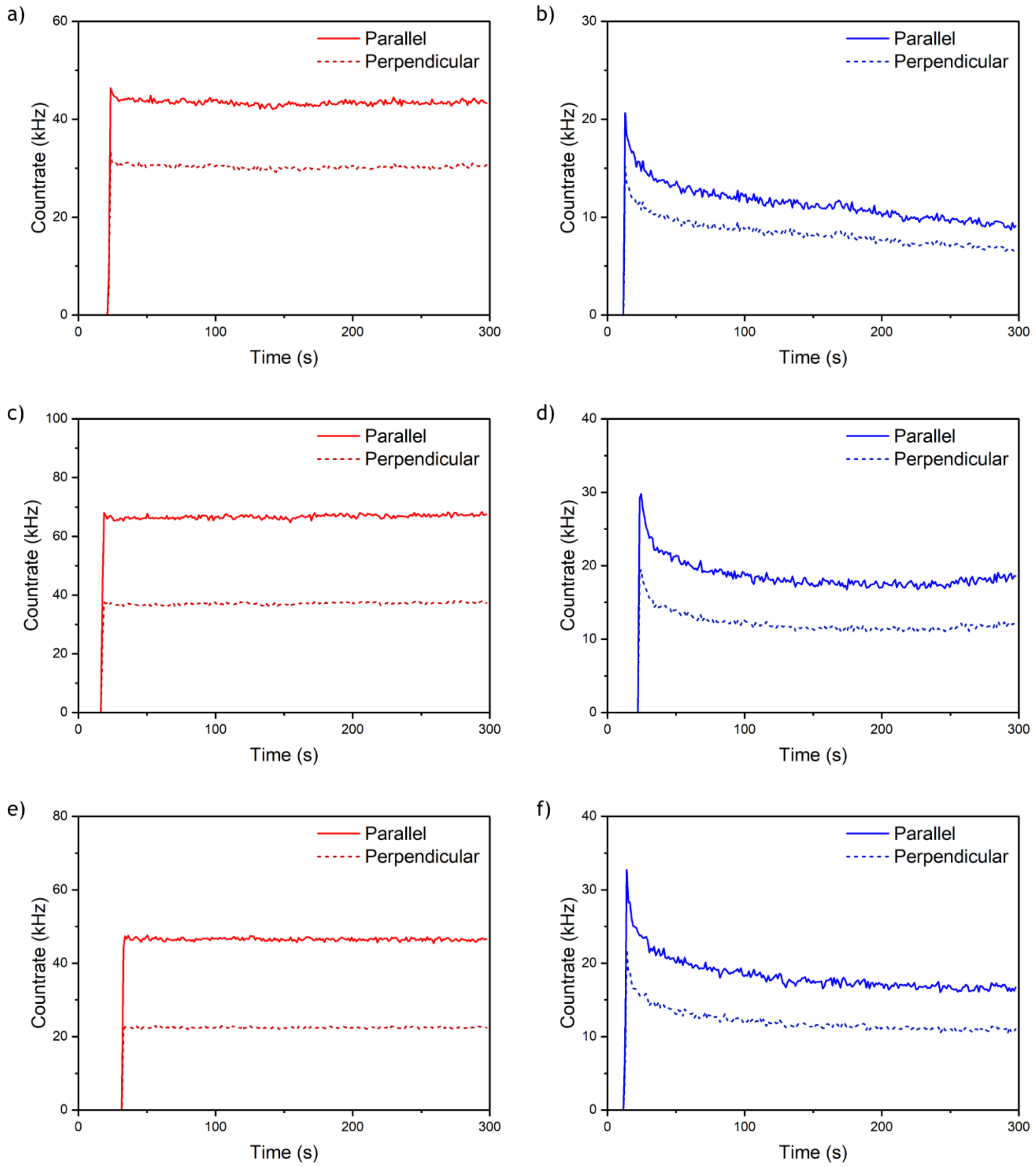

Figure S8. Comparison of the photostability of ABN containing oligonucleotides under one- and two-photon excitation. **a, b)** Countrate traces of GXT under two-photon **(a)** and one-photon **(b)** excitation. **c, d)** Countrate traces of GXT(A) under two-photon **(c)** and one-photon **(d)** excitation. **e, f)** Countrate traces of GXT(G) under two-photon **(e)** and one-photon **(f)** excitation. For the two-photon measurements the sample concentration was 100 nM and the excitation power was 11 mW. Samples were diluted to 10 nM for the one-photon measurements and the excitation power was 300 µW.

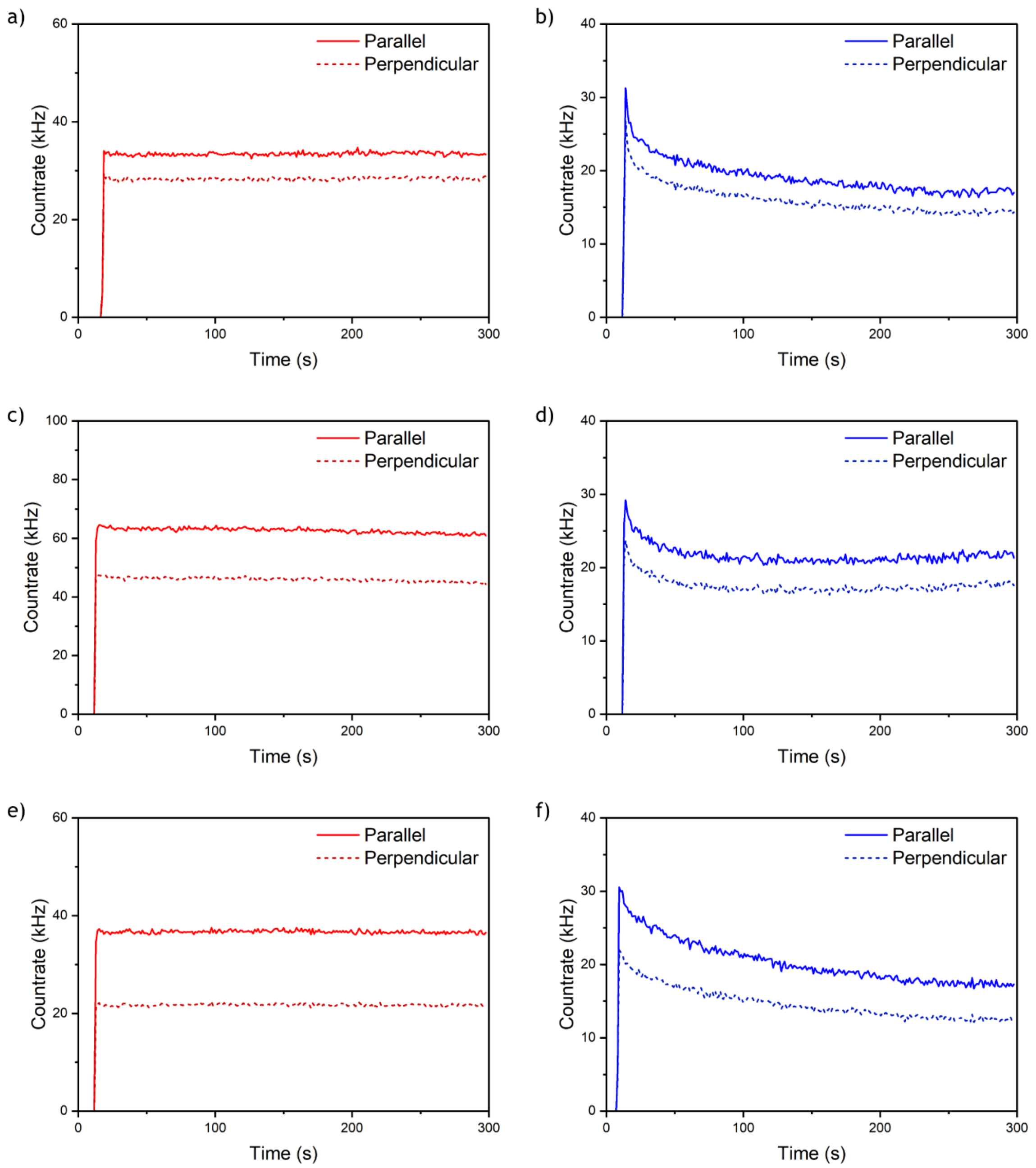

Figure S9. Comparison of the photostability of ABN containing oligonucleotides under one- and two-photon excitation. **a, b)** Countrate traces of AXA under two-photon **(a)** and one-photon **(b)** excitation. **c, d)** Countrate traces of AXA(A) under two-photon **(c)** and one-photon **(d)** excitation. **e, f)** Countrate traces of AXA(G) under two-photon **(e)** and one-photon **(f)** excitation. For the two-photon measurements the sample concentration was 100 nM and the excitation power was 11 mW. Samples were diluted to 10 nM for the one-photon measurements and the excitation power was 300 µW.

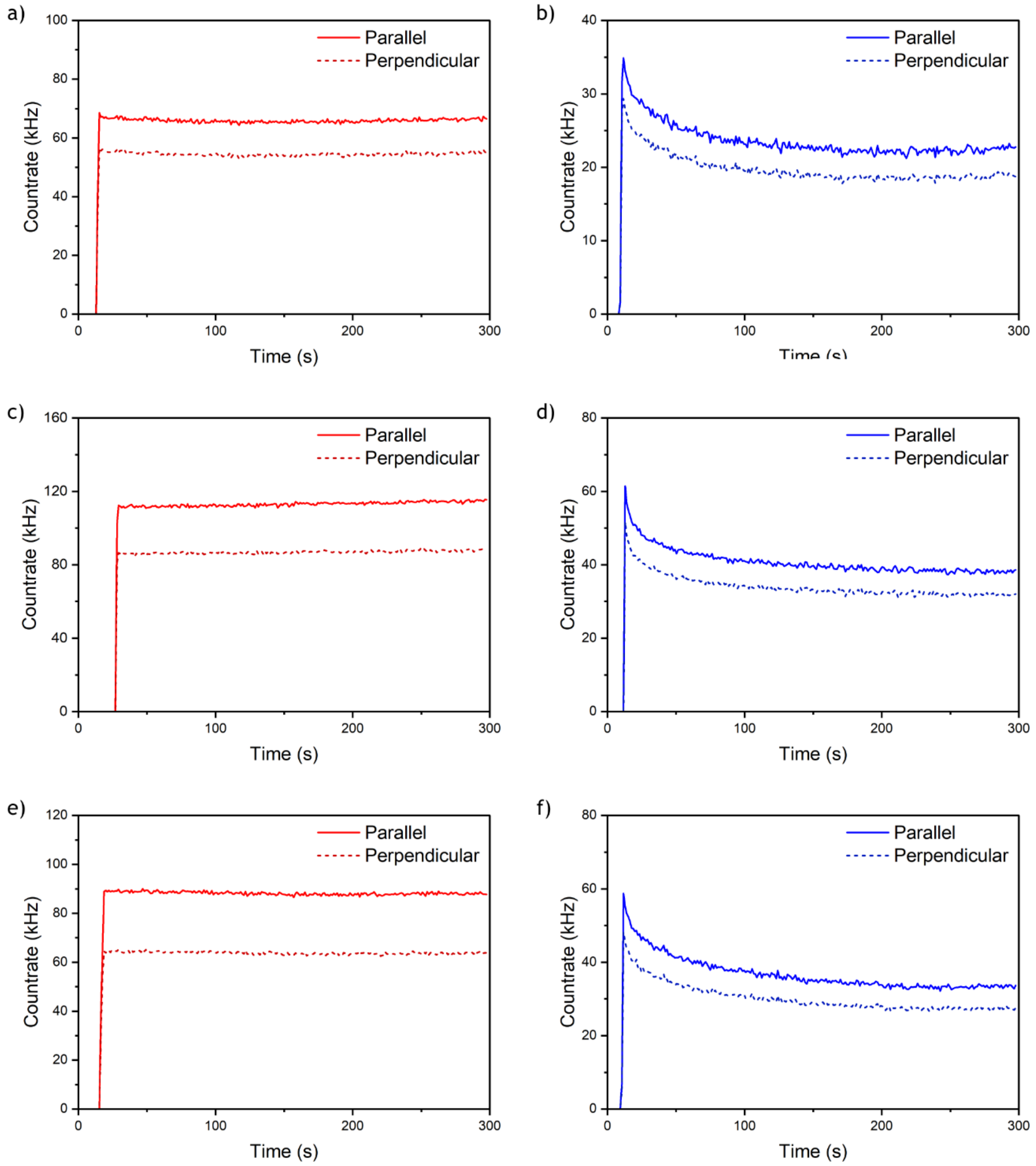

Figure S10. Comparison of the photostability of ABN containing oligonucleotides under one- and two-photon excitation. **a, b)** Countrate traces of GXC under two-photon **(a)** and one-photon **(b)** excitation. **c, d)** Countrate traces of GXC(A) under two-photon **(c)** and one-photon **(d)** excitation. **e, f)** Countrate traces of GXC(G) under two-photon **(e)** and one-photon **(f)** excitation. For the two-photon measurements the sample concentration was 100 nM and the excitation power was 11 mW. Samples were diluted to 10 nM for the one-photon measurements and the excitation power was 300 µW.

5. References

1. Samaan, G. N., et al., Single-molecule fluorescence detection of a tricyclic nucleoside analogue. *Chem. Sci.* **2021,** *12*, 2623-2628.
2. Samaan, G. N.; Jimenez Salinas, A.; Bailie, A. E.; Grim, J.; Cizmic, J. M.; Jones, A. C.; Lee, Y.; Purse, B. W., Single-molecule detection of oligonucleotides using the fluorescent nucleobase analogue ABN. *Chem. Sci.* **2025,** *16*, 4866-4875.
3. Chen, Y.; Müller, J. D.; So, P. T. C.; Gratton, E., The Photon Counting Histogram in Fluorescence Fluctuation Spectroscopy. *Biophys. J.* **1999,** *77* (1), 553-567.
4. Brand, L.; Eggeling, C.; Zander, C.; Drexhage, K. H.; Seidel, C. A. M., Single-molecule identification of Coumarin-120 by time-resolved fluorescence detection: Comparison of one- and two-photon excitation in solution. *J. Phys. Chem. A* **1997,** *101* (24), 4313-4321.
